# Aldehyde dehydrogenases contribute to skeletal muscle homeostasis in healthy, aging, and Duchenne muscular dystrophy patients

**DOI:** 10.1002/jcsm.12557

**Published:** 2020-03-10

**Authors:** Jessy Etienne, Pierre Joanne, Cyril Catelain, Stéphanie Riveron, Alexandra Clarissa Bayer, Jérémy Lafable, Isabel Punzon, Stéphane Blot, Onnik Agbulut, Jean‐Thomas Vilquin

**Affiliations:** ^1^ Sorbonne Université, INSERM, AIM, Centre de Recherche en Myologie, UMRS 974, AP‐HP Hôpital Pitié Salpêtrière Paris France; ^2^ Sorbonne Université, CNRS, INSERM, Institut de Biologie Paris‐Seine, IBPS, UMR 8256 Biological Adaptation and Ageing Paris France; ^3^ Université Paris‐Est Créteil, INSERM, Institut Mondor de Recherche Biomédicale, IMRB, École Nationale Vétérinaire d'Alfort, ENVA, U955‐E10 Maisons‐Alfort France; ^4^ Department of Bioengineering and QB3 Institute University of California Berkeley CA USA

**Keywords:** Aging, Aldehyde dehydrogenase, Dog model, Duchenne muscular dystrophy, Human, Skeletal muscle, Myogenic, Non‐human primate

## Abstract

**Background:**

Aldehyde dehydrogenases (ALDHs) are key players in cell survival, protection, and differentiation via the metabolism and detoxification of aldehydes. ALDH activity is also a marker of stem cells. The skeletal muscle contains populations of ALDH‐positive cells amenable to use in cell therapy, whose distribution, persistence in aging, and modifications in myopathic context have not been investigated yet.

**Methods:**

The Aldefluor® (ALDEF) reagent was used to assess the ALDH activity of muscle cell populations, whose phenotypic characterizations were deepened by flow cytometry. The nature of ALDH isoenzymes expressed by the muscle cell populations was identified in complementary ways by flow cytometry, immunohistology, and real‐time PCR *ex vivo* and *in vitro*. These populations were compared in healthy, aging, or Duchenne muscular dystrophy (DMD) patients, healthy non‐human primates, and Golden Retriever dogs (healthy vs. muscular dystrophic model, Golden retriever muscular dystrophy [GRMD]).

**Results:**

ALDEF^+^ cells persisted through muscle aging in humans and were equally represented in several anatomical localizations in healthy non‐human primates. ALDEF^+^ cells were increased in dystrophic individuals in humans (nine patients with DMD vs. five controls: 14.9 ± 1.63% vs. 3.6 ± 0.39%, *P* = 0.0002) and dogs (three GRMD dogs vs. three controls: 10.9 ± 2.54% vs. 3.7 ± 0.45%, *P* = 0.049). In DMD patients, such increase was due to the adipogenic ALDEF^+^/CD34^+^ populations (11.74 ± 1.5 vs. 2.8 ± 0.4, *P* = 0.0003), while in GRMD dogs, it was due to the myogenic ALDEF^+^/CD34^−^ cells (3.6 ± 0.6% vs. 1.03 ± 0.23%, *P* = 0.0165). Phenotypic characterization associated the ALDEF^+^/CD34^−^ cells with CD9, CD36, CD49a, CD49c, CD49f, CD106, CD146, and CD184, some being associated with myogenic capacities. Cytological and histological analyses distinguished several ALDH isoenzymes (ALDH1A1, 1A2, 1A3, 1B1, 1L1, 2, 3A1, 3A2, 3B1, 3B2, 4A1, 7A1, 8A1, and 9A1) expressed by different cell populations in the skeletal muscle tissue belonging to multinucleated fibres, or myogenic, endothelial, interstitial, and neural lineages, designing them as potential new markers of cell type or of metabolic activity. Important modifications were noted in isoenzyme expression between healthy and DMD muscle tissues. The level of gene expression of some isoenzymes (ALDH1A1, 1A3, 1B1, 2, 3A2, 7A1, 8A1, and 9A1) suggested their specific involvement in muscle stability or regeneration *in situ* or *in vitro*.

**Conclusions:**

This study unveils the importance of the ALDH family of isoenzymes in the skeletal muscle physiology and homeostasis, suggesting their roles in tissue remodelling in the context of muscular dystrophies.

## 1 Introduction

Aldehyde dehydrogenases (ALDHs) constitute a large family of isoenzymes that forged their diversity throughout evolution.^1^ In human, 19 isoenzymes harbour different functions involved in ontogenesis, development, regeneration, and homeostasis.^1^, ^2^, ^3^, ^4^, ^5^, ^6^, ^7^ Most ALDHs detoxify cells from endogenic or xenogeneic aldehydes that result from catabolic reactions or oxidative stress by catalysation into corresponding carboxylic acids.^4^, ^7^ Strong cellular protections against oxidative stress are conferred directly by these activities or, indirectly, through the production of secondary messengers able to activate other detoxifying enzymes (e.g. activation of glutathione peroxidase by retinoic acid).^8^ Such functions are responsible for resistance against cytotoxicity, mutagenicity, genotoxicity, and carcinogenesis. They also protect against toxic accumulation of reactive aldehydes produced by lipid peroxidation, such as malonaldehyde and 4‐hydroxynonenal, and the aldehyde‐protein adducts, which are especially involved in sarcopenia and the general process of aging.^9^, ^10^ These catalytic functions are also responsible for the resistance against some chemotherapeutic agents and participate in the status of cancer stem cells ascribed to some ALDH‐positive cell populations.^11^, ^12^, ^13^, ^14^, ^15^, ^16^, ^17^

Some ALDH isoenzymes provide retinoic acid (ALDH1A1, 1A2, 1A3, 1B1, 3B1, and 8A1),^7^ and their coordinated expression is finely tuned during ontogenesis of several tissues,^18^ such as the skeletal muscles^19^, ^20^, ^21^, ^22^ and heart.^23^, ^24^ ALDH1A1 is involved in myogenesis,^25^, ^26^, ^27^, ^28^ maintaining myogenic progenitors in an undifferentiated stage *in vitro*.^29^ ALDH1A2 and ALDH1A3 are involved in cardiogenesis,^30^ and ALDH1B1 is involved in the formation of pancreatic progenitors.^31^, ^32^, ^33^ ALDH3B1 is involved in diethylaminobenzaldehyde (DEAB)‐insensitive retinoic acid synthesis in some cell types.^34^ ALDH8A1 is involved in retinaldehyde metabolism, specifically the 9‐*cis* retinal, and in oxidation of aliphatic aldehydes and glutaraldehyde. ALDH1A2, 1A3, 3B1, and 8A1 especially metabolize aldehydes derived from lipid peroxidation.^2^, ^35^

Several isoenzymes are involved in other metabolic pathways. ALDH1L1 encodes the formyltetrahydrofolate dehydrogenase and is involved in neurulation and in neural and glial stem cells.^36^, ^37^ ALDH2 metabolizes acetaldehyde, and several mutations trigger intolerance to alcohol.^38^ ALDH2 detoxifies aldehydes derived from lipid peroxidation.^2^ ALDH2 is also involved in the metabolism of nitric oxide and plays roles in vascular adaptation, reactivity, and protection against ischaemia.^38^ ALDH3A2 is involved in the oxidation of fatty aldehydes and in stabilization of cellular lipid membranes. ALDH5A1 is involved in catabolism of gamma‐aminobutyric acid.^2^ ALDH7A1 is involved in the formation of zebra fish eyes and fins^39^ and scavenges peroxidized lipids,^40^ semialdehydes, acetaldehyde, and benzaldehyde. ALDH9A1 catalyses the oxidation of betaine and the synthesis of gamma‐aminobutyric acid.^2^, ^11^ ALDH isoenzymes, either alone or as a family of complementary agents, are therefore important regulators of several cell functions.

The fluorescent Aldefluor® (ALDEF) reagent identifies cell populations displaying ALDH activity, and it is widely used to identify stem cell populations from various tissues,^41^, ^42^, ^43^, ^44^, ^45^, ^46^, ^47^ including the skeletal muscle.^27^, ^28^, ^29^, ^48^ Upon oxidation, ALDEF becomes hydrophilic and is trapped within cells, which can be discriminated using flow cytometry or fluorescence microscopy. Previously, we described SSC^lo^/ALDEF^br^ cells extracted from dissociated biopsies of human skeletal muscles,^48^ and we distinguished two main sub‐populations according to the co‐expression of CD34 marker. ALDEF^+^/CD34^−^ cells developed *in vitro* as a population of CD56^+^ myoblasts were able to form myotubes and participated efficiently in muscle regeneration *in vivo* in immunodeficient mice, while ALDEF^+^/CD34^+^ cells harboured adipogenic and osteogenic capacities suggestive of a fibro‐adipogenic nature.^48^, ^49^, ^50^ The myogenic capacities of ALDEF^+^/CD34^−^ cells, together with the documented resistance of ALDH^+^ cells to oxidative stress, make them attractive candidates for cell therapy attempts to regenerate muscle tissues, especially in pathological contexts such as Duchenne muscular dystrophy (DMD).^27^, ^28^, ^29^, ^48^, ^51^, ^52^, ^53^ However, the persistence of ALDEF^+^ cell populations with aging, or their modulations in DMD, remains to be addressed, as several progenitors are reputed to decrease under these conditions.^54^, ^55^, ^56^ The exact nature of isoenzymes able to metabolize ALDEF is partly unknown, and most studies of muscle tissue focused on ALDH1A1 leaving unexplored the whole panel of ALDH isoenzymes expressed in parallel by muscle cells *in vivo* and *in vitro*.

In this study, we first assessed the persistence of ALDEF‐positive cell populations harvested from healthy patients of different ages. Then, to study a potential role in pathological context, we investigated the proportions of ALDEF^+^ cells in muscle samples of DMD patients and healthy donors, and of the suitable preclinical model, the Golden Retriever dog (Golden retriever muscular dystrophy [GRMD]).^57^ While no significant variation was observed with aging, important qualitative and quantitative changes were observed in those dystrophic tissues. We also associated several extracellular markers with ALDEF‐positive sub‐populations in these physiological contexts. In a second step, we identified the isoenzymes expressed *ex vivo* upon dissociation of muscle tissues and finally *in vitro* in both proliferation and differentiation, using flow cytometry, immunohistology, and semi‐quantitative PCR. Several isoenzymes were found associated with distinct cell types in the muscle tissue and may constitute potential new cellular markers. Taken together, our results suggest that several ALDH isoenzymes are expressed by myogenic and non‐myogenic cells, constituting new phenotypic or metabolic markers, and they underline quantitative and qualitative variations in dystrophic condition.

## 2 Materials and Methods

### 2.1 Biological samples

Human skeletal muscle samples were obtained as post‐surgical *res nullius* via the Tissue Bank for Research (Myobank‐AFM of Myology Institute, authorization no. AC‐2013‐1868), in agreement with the French bioethical law (Law no. 94‐654 of 29 July 1994, modified 22 January 2002; Ethics Committee number BB‐0033‐00012, norma NF S‐96‐900) upon informed and signed consent of the donors. The healthy donors were adults, they had no clinical signs of muscular disease, and they underwent uneventful hip surgery allowing harvesting *tensor fasciae latae* (TFL) samples. Men and women were equally represented. Hip surgery is typically performed as a consequence of hip aging; therefore, the number of biopsies from young donors was lower than that from older patients. Samples were provided in transport medium (Dulbecco's modified Eagle medium [DMEM] supplemented with gentamycin) for use as fresh tissue for dissociation and culture and used within 24 h after extraction. Samples were also snap frozen in isopentane cooled in liquid nitrogen for immunohistochemical analysis or snap frozen in liquid nitrogen for analysis of RNA expression. Pathological paravertebral samples were obtained from young boys presenting with DMD, aged 13–16 years and undergoing spinal orthosis surgery. Healthy controls for this group were young patients of both genders presenting with idiopathic scoliosis. Human liver and kidney samples were provided snap frozen in liquid nitrogen upon necropsia by the Department of Neuropathology R. Escourolle.

Samples from GRMD and healthy dogs (*biceps femoris*) were provided by École Nationale Vétérinaire (Alfort). Samples from non‐human primate (NHP) *Macaca fascicularis* were obtained from Institut du Cerveau et de la Moelle (Paris) and harvested from several distinct anatomical localizations. Dogs and NHP were obtained at the time of sacrifice of these animals involved in unrelated protocols, and none of these animals were sacrificed for the purpose of our study.

### 2.2 Muscle dissociations

The human muscle biopsies were processed within 24 h after collection.^58^ They were sliced, finely minced, and digested for 1 h at 37°C using 0.2% type II collagenase (Worthington) in DMEM (Invitrogen) supplemented with 10% defined foetal bovine serum (DFBS; HyClone). Mechanical dissociation was completed by passage of the cell dissociate through a 10 mL pipette, and an 18G needle. The suspension was filtrated through 100 then 40 μm cell strainers (Becton‐Dickinson, BD). The resulting cell suspensions were centrifuged, washed, and frozen in a medium containing 10% dimethyl sulfoxide, 20% DFBS, and 70% DMEM for later use. NHP and dog muscle biopsies were dissociated within 12 h after collection.

### 2.3 Cell cultures and expansion

Upon being thawed, the dissociated cells were washed once in phosphate‐buffered saline (PBS) supplemented with 2% DFBS and then seeded in 25 cm^2^ flasks (100–200 mg of the initial suspension) in the proliferation medium containing: 80% of modified MCDB120 (Molecular, Cellular and Development Biology) medium (custom‐made by HyClone‐Perbio), 20% DFBS, 25 μg/mL of gentamycin (Gibco), 10 ng/mL of human recombinant basic fibroblast growth factor (R&D Systems), and 1 μM of dexamethasone (Merck).^58^ After medium change on the following day, cultures were grown for 10 days to reach 60% confluence, and then cells were harvested by trypsinization and further expanded before reaching 80% confluence for three passages. Aliquots were analysed for ALDEF activity, CD56 expression, or differentiation studies.

### 2.4 ALDEF and extracellular markers characterization by flow cytometry

Following being thawed and washed, cell suspensions were incubated with the ALDEF substrate (Aldefluor®, 1 μM, Stemcell Technologies) for 20 min (human and NHP cells) or 45 min (dog cells), at 37°C. Controls were obtained by prior incubation of cells with 50 mM of the specific ALDEF inhibitor DEAB.^48^ Cells were centrifuged and kept on ice; surface antigens were detected by incubation with allophycocyanin‐labelled CD34 (BD) or phycoerythrin (PE)‐labelled CD9, CD10, CD29, CD31, CD36, CD44, CD47, CD49a, CD49b, CD49c, CD49d, CD49e, CD49f, CD56, CD61, CD71, CD105, CD106, CD140a, CD140b, CD143, CD146, CD184, and CD309 (3/100, 15 min, 4°C, BD). Cells were analysed using FACSCalibur (BD) and the CellQuest Software (2.10^3^ to 10^4^ events analysed, owing to the small size of the biopsies). Non‐specific fluorescence was determined using negative isotype controls (BD).^47^ Data were analysed and plotted using GraphPad Prism.

### 2.5 Cell sorting and myogenesis differentiation assay

Aldefluor® labelling was used for cell sorting in combination with labelling of CD34. Populations were sorted using a FACSAria cell sorter (BD) on the basis of ALDEF activity, APC‐labelled CD34, and PE‐labelled CD9, CD10, CD49e, or CD56. Cells were seeded into 12‐well plates, grown for 1 week in proliferation medium, and expanded. When confluence was reached, differentiation was induced using DMEM supplemented with 10% horse serum (AbCys). Five days later, cells were fixed and permeabilized using methanol (10 min, −20°C). The expression of desmin in myoblasts and myotubes was assessed using anti‐desmin mAb (1/300, 1 h, room temperature [RT], Dako) followed by goat anti‐mouse IgG1 Ab (Alexa Fluor 568, 1/1000, 1 h, RT). Nuclei were labelled with DAPI in mounting medium (Vectashield + DAPI, AbCys). Images were observed using a Zeiss epifluorescence microscope, images were captured using a Sony CCD cooled camera and the Metaview® software, and final figures were made using Adobe Photoshop®.

### 2.6 Muscle subcellular localization of aldehyde dehydrogenase isoenzymes by immunohistofluorescence

Six‐micrometre transverse cryostat sections were prepared from samples frozen in isopentane and fixed and permeabilized in acetone (10 min, −20°C); and non‐specific labelling was blocked using 10% DFBS in PBS (30 min, RT). Human ALDH1A1, 1A2, 1A3, 1B1, 1L1, 2, 3A1, 3A2, 3B1, 3B2, 4A1, 5A1, 6A1, 7A1, 8A1, 9A1, and 18A1 were labelled using rabbit, goat, or mouse Ab (1/100–1/500, 2 h, RT, *Table* 1) followed by the secondary Ab linked to a fluorophore (Alexa Fluor 568, 1/300 in PBS, 30 min at 4°C). Then sections were incubated with a rabbit polyclonal anti‐laminin Ab (1/200, 1 h, RT, Dako), followed by goat anti‐rabbit Ab (Alexa Fluor 488, 1/400, 1 h, RT) to delineate skeletal muscle fibres. In situations where the first Ab was already produced in the rabbit Ab, we first coupled the anti‐laminin Ab using the Mix‐n‐Stain CF488A antibody labelling kit according to supplier instructions (Sigma) and incubated this stained product (1/300, 30 min, RT). Nuclei were labelled with DAPI in mounting medium (Vectashield, AbCys). Negative controls were obtained by omitting primary Abs. Sections were observed using a Zeiss fluorescence microscope, images were captured using a Sony CCD cooled camera, and the Metaview® software and final figures were made using Adobe Photoshop®.

**Table 1:** Antibodies used in this study

Isoenzyme	Histology: species, provider, reference	Flow cytometry, cytofluorescence: species, provider, reference
ALDH‐1A1	Goat, Everest EB05049; goat Abcam 9883	Rabbit, Abcam 23375
ALDH‐1A2	Goat, SC 22591	Rabbit, SC 367527
ALDH‐1A3	Rabbit, Abgent AP7847a	Rabbit, Abgent AP7847a
ALDH‐1B1	Goat, Abcam 103896	NT
ALDH‐1L1	Rabbit, Abcam 190298	NT
ALDH‐2	Rabbit, Abcam 108306	Rabbit, Abcam 108306
ALDH‐3A1	Mouse, SC 137168	Rabbit, SC 67309
ALDH‐3A2	Rabbit, Abcam 113111	Rabbit, Abcam 113111
ALDH‐3B1	Goat, SC 109191	Rabbit, Abgent AP8706c
ALDH‐3B2	Rabbit, Abcam 112527	Rabbit, Abcam 112527
ALDH‐4A1	Rabbit, Abcam 59011, 185208	Rabbit, Abcam 59011, 185208
ALDH‐5A1	Goat, SC 70004; Mouse SC 515022	Goat, SC 70004; Mouse SC 515022
ALDH‐6A1	Mouse, SC 271582	Mouse, SC 271582
ALDH‐7A1	Rabbit, Epitomics 2300‐S	Rabbit, Epitomics 2300‐S; Abcam 68192
ALDH‐8A1	Rabbit, SC 130686; FS PA5‐63125	Rabbit, SC 130686
ALDH‐9A1	Rabbit, Abgent AP7850a	Rabbit, Abgent AP7850a
ALDH‐18A1	Rabbit, FS PA5‐52955	NT

FS, Fisher Scientific; NT, not tested; SC, Santa Cruz.

### 2.7 Localization of aldehyde dehydrogenase isoenzymes in primary cell cultures

The cell layers were washed in PBS, fixed in paraformaldehyde (PFA) (4% in PBS, 10 min, RT), permeabilized using saponin (0.2% in PBS, 10 min, RT), rinsed in PBS, and incubated using the rabbit Ab directed against isoenzymes (*Table* 1) followed by the secondary goat anti‐rabbit Ab linked to a fluorophore (Alexa 568, 1/300 in PBS, 30 min, RT). Simultaneously, cells were incubated with mouse anti‐desmin Ab (1/300, 1 h, RT, Dako), followed by goat anti‐mouse IgG1 Ab (Alexa Fluor 488, 1/400, 1 h, RT) to define the myogenic structures. Nuclei were labelled using DAPI. Images were captured using a Sony CCD cooled camera and the Metaview® software, and final figures were made using Adobe Photoshop®.

### 2.8 Characterization of isoenzymes by flow cytometry

After being thawed and washed, cell suspensions were fixed in PFA (4% PBS, 10 min, RT), permeabilized in saponin (0.2% in PBS, 10 min, RT), then rinsed in PBS, and incubated with ALDH isoenzymes, followed by the corresponding secondary Ab linked to fluorescein isothiocyanate (FITC) (1/300 in PBS, 30 min, RT). Some Abs were not available at the time of these experiments or not indicative under these conditions. Cells were centrifuged and analysed by flow cytometry (FACSCalibur, BD) using the Cell Quest Software (1.10^3^ to 10^4^ events analysed); results were plotted and analysed using GraphPad Prism.

### 2.9 Messenger RNA expression analysis by quantitative PCR

The primers were designed using the OLIGO Primer Analysis Version 7, with a size between 18 and 23 nucleotide and annealing temperature of 60°C (*Table* 2). Primers were designed containing an intron sequence for specific cDNA amplification, and reactions were performed with appropriate negative template‐free controls.

**Table 2:** List of human primers used in the study

	Primers
h/ALDH1A1	F: GGCCCTCAGATTGACAAGGA
	R: ATGATTTGCTGCACTGGTCC
h/ALDH1A2	F: AAGCTGGGACTGTTTGGATCA
	R: TACTCCCGCAAGCCAAATTC
h/ALDH1A3	F: ACGGTCTGGATCAACTGCTA
	R: CCGTCCGATGTTTGAGGAAG
h/ALDH1B1	F: AGACGGTCACCATCAAGGTT
	R: AGCATTCGTCAAGGTGGTTG
h/ALDH1L1	F: AGACCTTCCGCTACTTTGCT
	R: ATGATGCCACAAACCCCAAC
h/ALDH1L2	F: GCTTTCCAAAGGGGGTCATC
	R: GCTAACAGCACAGCTCTTCAT
h/ALDH2	F: GGGAGAGCCAACAATTCCAC
	R: CCACTCCCCGACATCTTGTA
h/ALDH3A1	F: ATCGCCTGGGGGAAATTCAT
	R: AGTCCCGGGATTTCTTAGCA
h/ALDH3A2	F: TTGGTACTTCCCAGGGCTAC
	R: GGTCAAGTCCTTGAGTCCCA
h/ALDH3B1	F: CTTTTGGAGGAGTGGGTGC
	R: GCGTTGAGCTTCTCCATCC
h/ALDH3B2	F: CCACTACCCACCCTATACCG
	R: GTGAGTTGGGAGCATAAGCC
h/ALDH4A1	F: AGCCTCTGGAACCAATGACA
	R: CACCTGGACGGACAGACAG
h/ALDH5A1	F: GACGAAGCACCTTCCTTTCC
	R:ATAGCTTCCCAGTGGCTCAA
h/ALDH6A1	F: TCACCGCTTTTGGTTGATCC
	R: TGTGGGATAAAAGAGGGGCT
h/ALDH7A1	F: GGTTGCCCTTGGATCTGTTC
	R: TGAACTTTGCCCAGCTCTCT
h/ALDH8A1	F: GCAGGGAACACTGTGATAGC
	R:GGTGGAACACCTGCTTTATCC
h/ALDH9A1	F: AGACGACATGACCTGTGTGA
	R: CCGTTGGATGTCCCTGGTAA
h/ALDH16A1	F: TTCGGATCAGCCCAGGGTTC
	R: TCAGGCATCAGTCCCCCATA
h/ALDH18A1	F: CCTGCAGGGGGTAAATGTTATT
	R: TCACAGACTGCTGATCTCCG
h/SRP72	F: TGCTGCTGTGTTTGACTCTG
	R: GCAGCACCCCATTTCTTTCT

Total mRNA was isolated from snap‐frozen tissue biopsies (100 mg) or cell pellets (100 000 cells at least) using TRIzol. Concentration and purity were evaluated with NanoDrop® ND‐1000 Spectrophotometer (Labtech).

First‐strand cDNA was synthetized from 250 ng of total mRNA with random hexamer primers using the RevertAid first‐strand cDNA synthesis kit (Thermo Scientific).

Quantitative PCR was carried out in 384‐well plates on cDNA products diluted to 1/25 in duplicate using SYBR Green (Roche) on a LightCycler® 480 (Roche) with the following parameters: initial denaturation step (95°C, 5 min), then 40 cycles composed of denaturation step (95°C, 30 s), and annealing/extension steps (60°C for 15 s, 72°C for 15 s). Srp72 was used as the reference gene. The ratio between the amounts of a target gene and the endogenous reference gene was determined. The amplification efficiencies between genes were compared by preparing a dilution series for genes from cDNA samples. Each dilution series was amplified in RT‐Q‐PCR, and the *C*_T_ values obtained were used to construct standard curves for targets. The amplification efficiency (*E*) for each target was calculated according to the following equation: *E* = 10^(−1/*S*)^ − 1 (where *S* = slope of the standard curve). Results were plotted, analysed, and compared using GraphPad Prism.

### 2.10 Statistical methods

**Figure 1:**
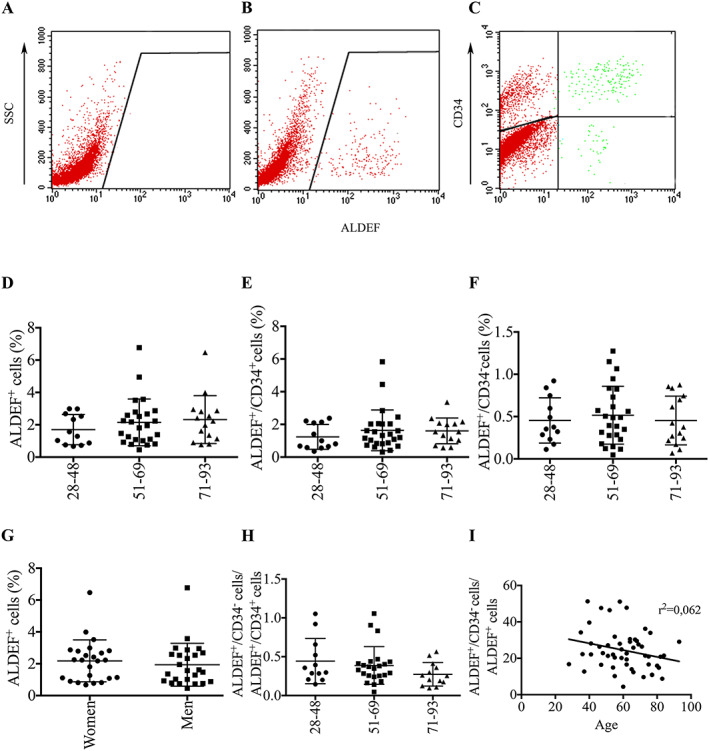
Flow cytometry characterization of ALDEF^+^/CD34^+^ and ALDEF^+^/CD34^−^ populations of cells extracted from patients of different ages. Dissociated cells from human TFL muscle biopsies were incubated with ALDEF substrate in the presence of DEAB inhibitor (A) or in its absence (B) and then with APC‐labelled anti‐CD34 (C). Representative cytograms are shown. The expression of CD34 can be represented as a function of ALDEF^+^ cells, providing an upper right quarter containing the ALDEF^+^/CD34^+^ cells and a lower right quarter containing the ALDEF^+^/CD34^−^ cells, in which the proportions can be estimated (C). Three cohorts of patients have been designed according to their ages (24–48, 51–69, and 71–93 years), and results have been plotted accordingly. No significant difference was observed between cohorts in the percentages of total ALDEF (D), of ALDEF^+^/CD34^+^ (E), or ALDEF^+^/CD34^−^ (F), nor in the ratio of these populations (H), although a non‐significant tendency was observed towards a decrease (I). No significant difference was demonstrated between the cohorts of men and women (G). Data are percentages (mean, SD) of cells positive for the indicated marker. One‐way ANOVA with Tukey's multiple comparisons was used for statistics in graphs (D), (E), (H), and (I) and unpaired *t*‐test for graph (G).

**Figure 2:**
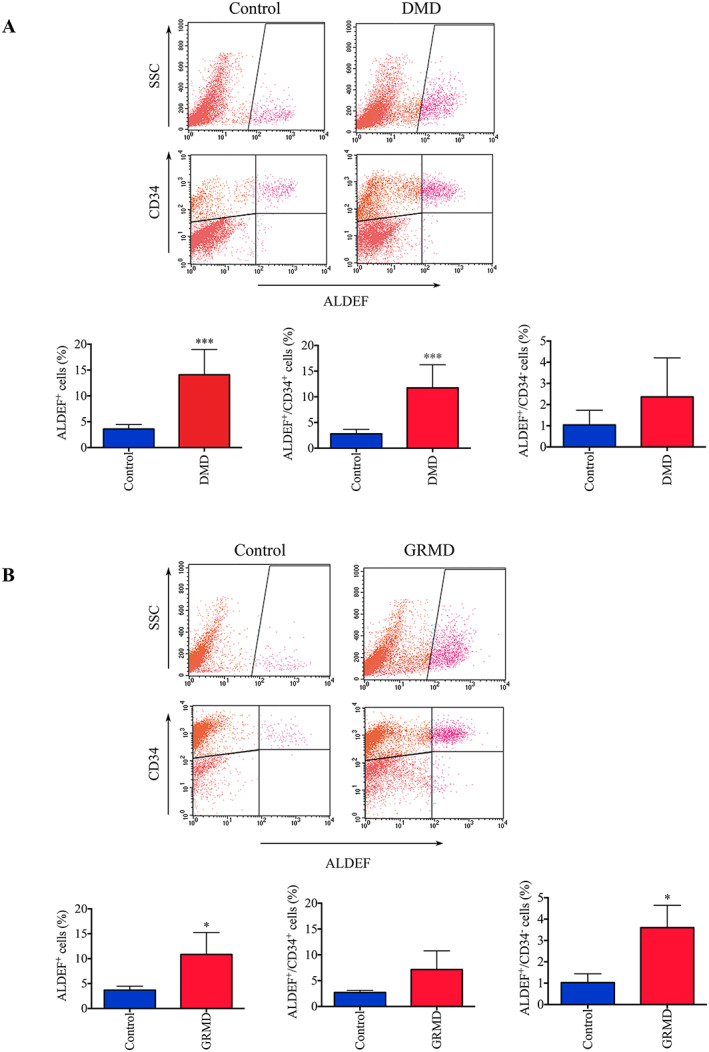
Flow cytometer evaluation of cell populations extracted from healthy and DMD patients, and from control and GRMD dogs. Cells were dissociated from muscle biopsies harvested from healthy (*n* = 9) and DMD patients (*n* = 5) (A) or control (*n* = 3) and GRMD dogs (*n* = 3) (B). Cells were incubated with ALDEF substrate and APC‐labelled anti‐CD34 allowing to distinguish ALDEF^+^/CD34^+^ and ALDEF^+^/CD34^−^ populations. Representative cytograms are shown; mean and SD are plotted on histograms (right panels). Total ALDEF^+^ cells are increased in DMD patients (*P* = 0.0002) and GRMD dogs (*P* = 0.0499), the ALDEF^+^/CD34^+^ population is significantly increased in DMD patients (*P* = 0.0003) but not in GRMD dogs, while the ALDEF^+^/CD34^−^ population is significantly increased in GRMD dogs (*P* = 0.0165) but not in DMD patients. Unpaired *t*‐test with Welch's correction was applied for control vs. DMD patients and unpaired *t*‐test for control vs. GRMD dogs.

**Figure 3:**
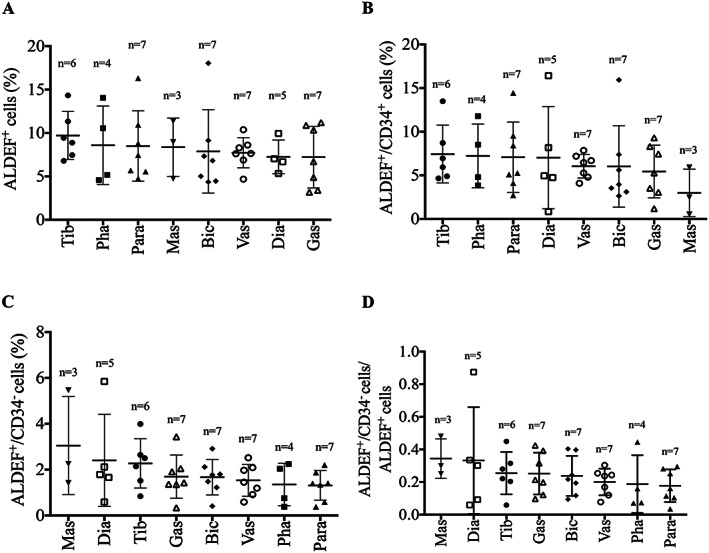
Flow cytometer evaluation of cell populations extracted from distinct anatomic territories. Cells were dissociated from muscle biopsies harvested from several territories of NHP (macaques) of similar ages (*n* = 3–7). Cells were incubated with ALDEF substrate and then with APC‐labelled anti‐CD34, allowing to distinguish the macaque cell populations. The contents in total ALDEF^+^ (A), in ALDEF^+^/CD34^+^ (B), in ALDEF^+^/CD34^−^ (C) populations, or their ratios (D) were compared and presented variations, and no significant difference between muscle groups could be established. *n* indicates the number of samples for each muscle group. One‐way ANOVA with Tukey's multiple‐comparison test.

**Figure 4:**
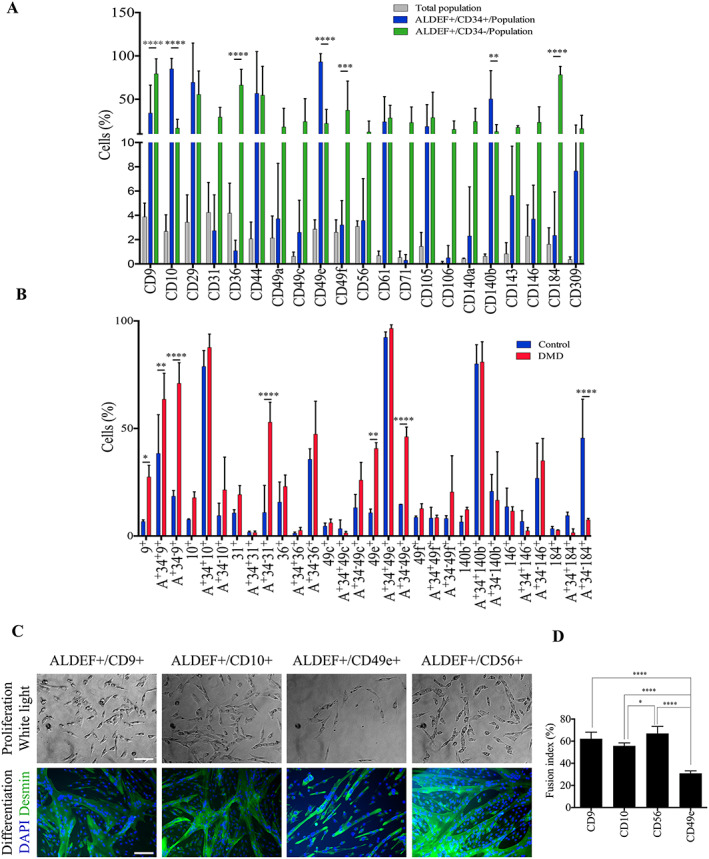
Identification of markers associated with ALDEF^+^ cell populations. Dissociated cells from human control TFL muscle biopsies were incubated with ALDEF substrate and then with APC‐labelled anti‐CD34 antibody and a second PE‐labelled marker. Histograms and data are percentages (mean, SD) of positive cells for the indicated markers. (A) Cell analyses from TFL muscles of healthy controls (*n* = 7). The proportion of a dedicated marker among the whole content of mononucleated cells is presented in grey. The percentages of cells expressing a dedicated marker within the ALDEF^+^/CD34^+^ population and within the ALDEF^+^/CD34^−^ population are presented in blue and in green, respectively. This representation suggests that, for example, CD9 and CD184 are especially co‐expressed with ALDEF^+^/CD34^−^ cells from skeletal muscles. (B) Comparison of some selected markers expressed by cells extracted from healthy (blue, *n* = 4) and DMD (red, *n* = 4) paravertebral muscles. For each marker, the three sub‐populations are individualized (all cells, ALDEF^+^/CD34^+^, and ALDEF^+^/CD34^−^). Significant differences were observed regarding DMD cell populations expressing CD9, CD31, and CD49e and healthy cell populations expressing CD184. The myogenic differentiation capacities of cells selected on the basis of these markers is compared (C and D). NHP muscle cells (*n* = 4 animals) were incubated with ALDEF substrate and then with APC‐labelled anti‐CD34 antibody and a second PE‐labelled marker—CD9 (*n* = 4), CD10 (*n* = 2), CD56 (*n* = 4), or CD49e (*n* = 4)—and then sorted using a FACSDiva. The selected populations were grown in culture in proliferating medium for two passages (C, left) and then differentiated, fixed, and labelled for desmin expression (green) and DAPI staining (blue) (C, right). Fusion indexes were calculated (*n* = 4 fields per well) (D). Cells selected on CD9 and CD56 were the most myogenic, while cells selected on CD49e were significantly less myogenic (*P* < 0.01) (bottom panel). Data are presented as mean and SD. Two‐way ANOVA with Sidak's multiple‐comparison test was applied (A and B) and one‐way ANOVA with Tukey's multiple‐comparison test (C; *P ≤ 0.05, **P ≤ 0.01, ***P ≤ 0.001, and ****P ≤ 0.0001).

All plotted dots are independent biological replicates (individual human or non‐human primates), and all statistical tests were performed on GraphPad Prism software. Single comparisons were made using unpaired *t*‐test (*Figures* 1G and 2B) or unpaired *t*‐test with Welch's correction (*Figure* 2A), while multiple comparisons were performed using two‐way analysis of variance (ANOVA) with Sidak's multiple‐comparison tests (*Figure* 4A and 4B) or Tukey's multiple‐comparison tests (*Figures* 1D–F, 1H, 1I, 3A–D, and 4C). *P* values are reported in the figures.

## 3 Results

### 3.1 Assessment of cells metabolizing ALDEF

#### 3.1.1 ALDEF^+^ cells persist through aging

Muscle biopsies were collected from healthy patients undergoing hip replacement and were dissociated by mechanical and enzymatic treatments. The patients (25 women and 27 men, aged between 28 and 93 years) were divided into three experimental groups on the basis of age—28–48 (*n* = 12), 51–69 (*n* = 25), and over 71 years (*n* = 15)—and the proportion of cells metabolizing ALDEF was analysed in the presence (*Figure* 1A) or absence (*Figure* 1B) of the inhibitor DEAB and in association with CD34 (*Figure* 1C). Variability was noted in the proportions of ALDEF^+^ cells, especially in the groups of patients aged >50 (*Figure* 1D–F), but the changes were not significant over the years whether ALDEF^+^ cells were associated with CD34 or not (*Figure* 1E, F). Additionally, no significant difference was noted according to gender (*Figure* 1G) or when split into its two components, ALDEF^+^/CD34^+^ and ALDEF^+^/CD34^−^. Finally, the ratio of the ALDEF^+^/CD34^−^ cells, considered as the most myogenic one, was slightly increased in the youngest patients but stable in those older than 50; however, the slope of the curve did not show a significant difference (*r* ^2^ = 0.062, *Figure* 1H, I).

#### 3.1.2 ALDEF^+^ cells in a dystrophic context

The stable situation observed earlier was drastically modified in muscle tissue prepared from DMD patients whose muscle structure and function are highly altered owing to adipo‐fibrotic infiltration. Paravertebral muscle biopsies from these young patients operated for orthopaedic spine surgery (*n* = 9; median age 15.4 years) were compared with those harvested from young healthy patients operated for idiopathic scoliosis (*n* = 5; median age 13.75 years). The percentage of ALDEF^+^ cells was dramatically increased in DMD patients, essentially owing to the increase in ALDEF^+^/CD34^+^ population (*Figure* 2A), which was mainly considered as non‐myogenic; meanwhile, the percentage of ALDEF^+^/CD34^−^ population remained stable

GRMD dogs, a large animal model of DMD, develop the progressive myopathy on both clinical and histological aspects owing to the absence of dystrophin. Here again, the percentage of ALDEF^+^ cells extracted from biceps femoris biopsies dramatically increased (*n* = 3) as compared with those of control dogs (*n* = 3). Surprisingly, we observed a combined increase in the ALDEF^+^/CD34^+^ and ALDEF^+^/CD34^−^ cell populations (*Figure* 2B), but it should be noted that GRMD dogs were young animals that still present ongoing muscle tissue remodelling at time of surgery. The increase in the ALDEF^+^/CD34^−^ population may reflect the ongoing regenerative activity and/or muscle remodelling at the time of biopsy collection, compared with that in DMD patients who suffer from fixed, established myopathy at their age (15.4 years).

#### 3.1.3 Anatomical origin does not influence the proportion of ALDEF^+^ cells

We next investigated whether differences in cell populations would be observed among anatomical territories. For this purpose, we compared the populations of ALDEF^+^ cells, associated or not with CD34, from different muscles of NHP *M. fascicularis*: *diaphragm* (Dia), *pharyngeal constrictor* (Pha), *masseter* (Mas), *paravertebral multifidus spinae* (Para), *biceps brachii* (Bic), *tibialis anterior* (Tib), *gastrocnemius* (Gas), and *vastus medialis* (Vas) (*Figure* 3A). The initial proportion of total ALDEF^+^ cells was higher than that observed in human and reached 8.51 ± 0.97%, and we only observed slight differences in richness between muscle groups (*Figure* 3B, C), which could be ranked, in decreasing mean proportion as follows: Tib > Pha > Para > Mas > Bic > Vas > Dia > Gas. The population of ALDEF^+^/CD34^+^ cells represented the majority, as in human patients (6.35 ± 1.68%, i.e. 75% of all ALDEF^+^). The populations of ALDEF^+^/CD34^−^ represented the minority (1.81 ± 0.58%, i.e. 25% of all ALDEF^+^) and were ranked as follows: Mas > Dia > Tib > Gas > Bic > Vas > Pha > Para. The embryologic origin of these muscles, i.e. somatic or facial, did not influence significantly the proportions of ALDEF^+^ cells; however, the muscle group containing the highest proportion of ALDEF^+^/CD34^−^ cells (Mas) is not a postural or locomotor muscle.

#### 3.1.4 Extracellular markers distinguished sub‐populations of ALDEF^+^ cells

ALDH activity represents intracellular, metabolic markers, which can be complemented by the study of associated extracellular markers. We analysed the presence of several extracellular antigens alone and/or in association with ALDEF^+^/CD34^+^ or ALDEF^+^/CD34^−^ clusters to identify in a reliable and comfortable manner the most myogenic over all cell populations. *Figure* 4A shows that the expression of several antigens may discriminate the populations (*n* = 7). Hence, the ALDEF^+^/CD34^+^ cells were frequently expressing CD10 (CALAA), CD49e (integrin alpha5), and CD140b (PDGF beta receptor), while the ALDEF^+^/CD34^−^ cells were preferentially associated with CD9 (MRP‐1), CD31 (PECAM), CD36 (PAS IV), CD49a (integrin alpha1), CD49c (integrin alpha3), CD49f (integrin alpha6), CD71 (transferrin receptor), CD106 (VCAM), CD140a (PDGF alpha receptor), CD146 (MCAM), and CD184 (CXCR4). Of note, ALDEF^+^/CD34^−^ cells were frequently associated with some classical myogenic markers (such as CD9, CD106, and CD184), but not all of them because a minority of cells express CD56 (NCAM). Finally, some markers were exclusive of one population, for example, CD29, CD44, CD47 (not shown), CD61, CD105, CD143, and CD309 were expressed by similar percentages of ALDEF^+^/CD34^+^ and ALDEF^+^/CD34^−^ cells.

The characterization was repeated with cell populations extracted from DMD patient biopsies (*Figure* 4B), focusing on the most discriminating markers identified earlier (CD9, CD10, CD31, CD36, CD49c, CD49e, CD49f, CD140b, CD146, and CD184). Differences were observed between control (*n* = 4) and DMD (*n* = 4) paravertebral biopsies. Total CD9^+^ and CD49e^+^ populations were increased in DMD biopsies. ALDEF^+^/CD34^−^ cell populations were mainly associated with CD9, CD31, CD36, CD49c, CD49e, CD49f, and CD146. The two populations ALDEF^+^/CD34^−^/CD31^+^ and ALDEF^+^/CD34^−^/CD49e^+^ were significantly increased in DMD biopsies as compared with control, and conversely, the ALDEF^+^/CD34^−^/CD184^+^ population was increased in control biopsies as compared with DMD.

#### 3.1.5 Myogenic capacities of ALDEF^+^ cell populations

Because some populations of ALDEF^+^ cells can be defined by the expression of specific extracellular markers, we sorted NHP ALDH‐expressing cells on the basis of extracellular markers associated with at least 30% of ALDEF^+^/CD34^+^ or ALDEF^+^/CD34^−^ cells. (i.e. CD9, CD10, CD49e, and CD56). Selected cells were grown, expanded once for amplification, and then committed to differentiation (*Figure* 4C). The proportions of myogenic cells and the fusion indexes were counted after desmin immunostaining (a cytoskeletal marker of myogenic cells, both myoblasts and myotubes). We observed that the cultures performed from CD49e^+^ cells were the less myogenic ones and contained the smallest number of myotubes. In contrast, the cultures associated with CD9^+^ and CD56^+^ were the most myogenic. Cultures performed from CD10^+^ cells presented intermediate values of myogenicity (*Figure* 4D).

#### 3.1.6 Expansion of ALDEF^+^ populations in primary cultures

**Figure 5:**
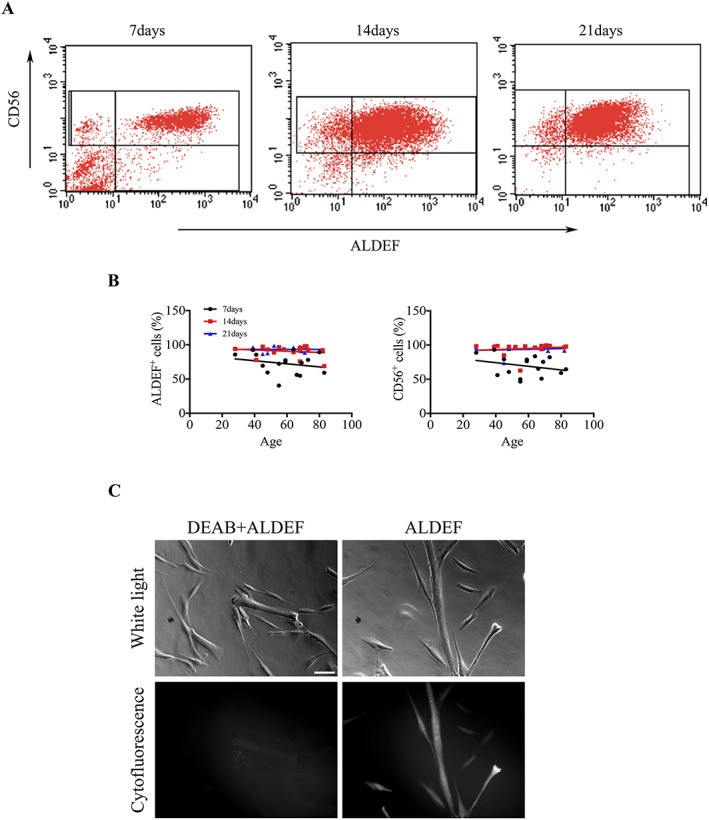
Expression of ALDH activity in culture. Cells were extracted from muscle biopsies of human donors of different ages and grown in culture in proliferating medium. Seven, 14, and 21 days after onset of cultures, ALDH expression was assessed using ALDEF, and the percentage of myogenic cells assessed using PE‐labelled anti‐CD56 antibody. (A) Increased proportions of cells express ALDEF and CD56 over time, and a few cells remain CD56^+^/ALDEF^−^. This behaviour is observed regardless of the ages of donors (B). The microscopic cytofluorescence observation of cultures following induction of cell differentiation indicates that ALDEF is also oxidized and accumulates in both myoblasts and myotubes (C).

We next analysed the long‐term ALDEF metabolism in human primary cultures upon expansion to third passage without selection, and we compared it with the classical evolution of the CD56 marker in the same conditions. The proliferation was obtained in a myogenic medium previously described.^58^ As soon as in first passage (7 days in culture), the majority of cells were ALDEF^+^, regardless of the age of donors, and this proportion was close to 100% in the second (14 days) and third (21 days) passages. These proportions paralleled the expression of CD56 (95% in the second passage) (*Figure* 5A, B). These results were confirmed by fluorescence microscopy (*Figure* 5C) showing the fluorescence emitted by myogenic cells in proliferation and by myotubes upon differentiation in the presence of ALDEF *in vitro*. These results indicate that the isoenzymes metabolizing ALDEF are expressed in culture and involved in myogenesis.

The exact nature of isoenzymes metabolizing ALDEF is unknown. ALDEF is a small soluble molecule that remains trapped in living cells, and as it is not a protein, it cannot be cross‐linked and fixed using classical protocols, hampering the co‐localization of ALDEF and other intracellular markers. To have a broader picture about the presence and roles of ALDH, we attempted their characterization using antibodies and gene expression analysis.

### 3.2 Characterization of aldehyde dehydrogenase isoenzymes

#### 3.2.1 Identification of the isoenzymes expressed by mononucleated cells extracted from biopsies

**Figure 6:**
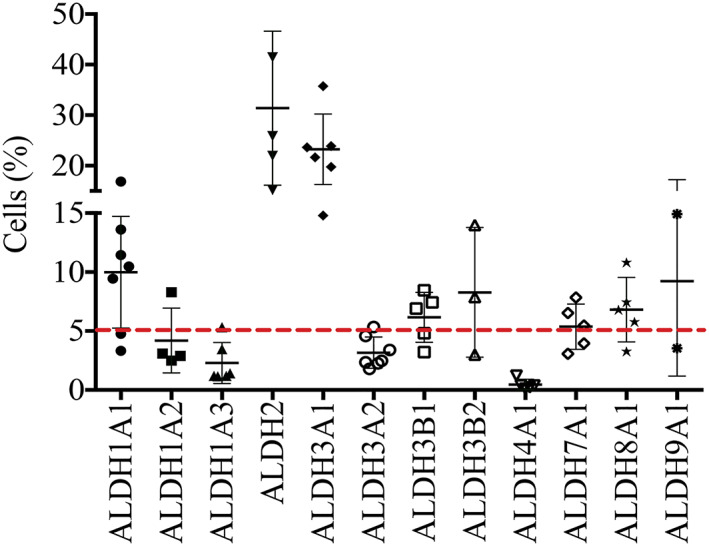
Identification of isoenzymes expressed by human freshly dissociated cells. Human cells were fixed using PFA and permeabilized using saponin, then incubated with antibodies directed against the ALDH isoenzymes indicated, then incubated with a secondary antibody labelled with FITC, and analysed by flow cytometry. Some isoenzymes were present in a high proportion of cells (up to 30% regarding ALDH2), while other isoenzymes were detected in smaller percentages of cells, with values around 5% but raising 10% (ALDH1A1 and ALDH9A1). A baseline was set arbitrarily at 5% to illustrate a maximum percentage of human cells detected using the ALDEF assay. This illustrates the potential discrepancy between the identification of the protein content using Ab, and the enzymatic activity detected by a functional assay. Data are presented as mean and SD (*n* = 2–7).

With the use of flow cytometry, antibodies identified most isoenzymes (*Figure* 6). From <1% (ALDH4A1) to >20% (ALDH2) of cells contained isoenzymes. Surprisingly, these values differ more or less from the number of cells able to metabolize ALDEF *in vitro*, which has been evaluated to 2–5%. Based arbitrarily on the proportion of cells stained by the Abs, a group of isoenzymes could be constituted in which <5% of the extracted cells were positive, gathering ALDH1A2, 1A3, 3A2, and 4A1. In the second group, >5% of the extracted cells were stained for ALDH1A1, 2, 3A1, 3B1, 3B2, 7A1, 8A1, or 9A1. Not all these isoenzyme‐containing cells were metabolizing the ALDEF, but these results call for different methodologies. Abs, which are directed against the ALDH proteins, identify more cells than does the quantification of ALDH enzymatic activity. Indeed, while ALDH2 and ALDH1A2 are thought to be detected by ALDEF,^14^ the proteins can undergo several post‐translational modifications (oxidation, phosphorylation, nitrosylation, and adduct formation), depending on cellular environment and subcellular localization, which modify their enzymatic activities.^59^

#### 3.2.2 Identification of the isoenzymes expressed by myogenic cells in culture

**Figure 7:**
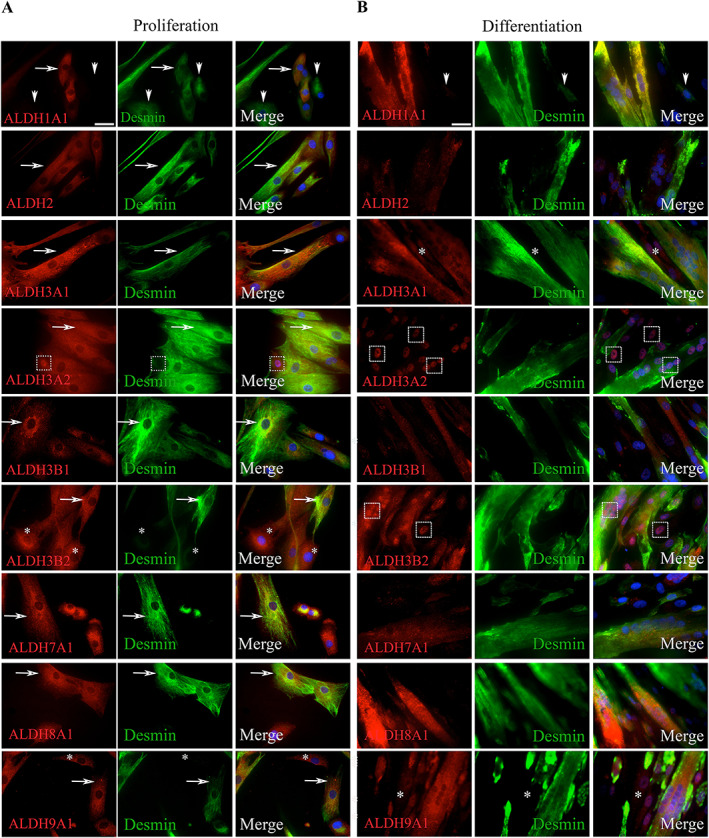
Immunocytological phenotyping of primary muscle cells in culture. Human cells were extracted from TFL muscles of healthy donors and grown in culture in proliferation medium (two passages, A) or in proliferation (two passages) and then in differentiation medium (4 days, B). Cells were fixed using PFA and permeabilized using saponin; and then incubated with antibodies directed against ALDH isoenzymes, and desmin; and then incubated with labelled secondary antibodies. Nuclei were stained using DAPI (blue). In proliferation (A), the number of cells expressing isoenzymes (red, first column) generally exceeded the number of cells expressing desmin (green, second column) (ALDH3A2, 3B2, 7A1, 8A1, and 9A1, arrows) except for 1A1 where some desmin^+^ cells were negative for the isoenzyme. ALDH3A2 stained strongly some nuclei. Following differentiation (B), myotubes were generally labelled with a stronger intensity than mononucleated cells (ALDH3A1, 3B1, and 9A1). The intensities of ALDH1A1 and 2 labelling were decreased as compared with cells in proliferation. Several nuclei strongly expressed ALDH3A2, suggesting a translocation from cytoplasm to nucleus. Original magnifications: ×40.

Human cells were grown in proliferation or in differentiation medium, fixed, and stained for the myogenic marker desmin, and for the isoenzymes showing previously the strongest expression (nine isoenzymes selected). We observed that during proliferation, ALDH isoenzymes labelled both myogenic and non‐myogenic cells (*Figure* 7A). None seemed completely restricted to non‐myogenic cells or to myogenic cells. Most presented a cytoplasmic staining, while others showed a nuclear (ALDH3A2) or perinuclear staining (ALDH3B1 and 3B2). During differentiation, ALDH isoenzymes were detected in the myotubes (*Figure* 7B) with a very weak (ALDH2 and 7A1) or strong intensity (ALDH3A1, 3B2, and 9A1).

Qualitative changes were noted between proliferating and differentiating cultures. Upon differentiation, ALDH1A1 was detected in myotubes but not in non‐myogenic cells; ALDH2 staining was dramatically reduced; ALDH3A2 was concentrated in nuclei; ALDH3B1 was detected only in myotubes. Changes in staining patterns and intensities suggest some roles for several isoenzymes in the course of differentiation *in vitro*.

#### 3.2.3 Histological localization of cell populations in healthy and Duchenne muscular dystrophy muscles

**Figure 8:**
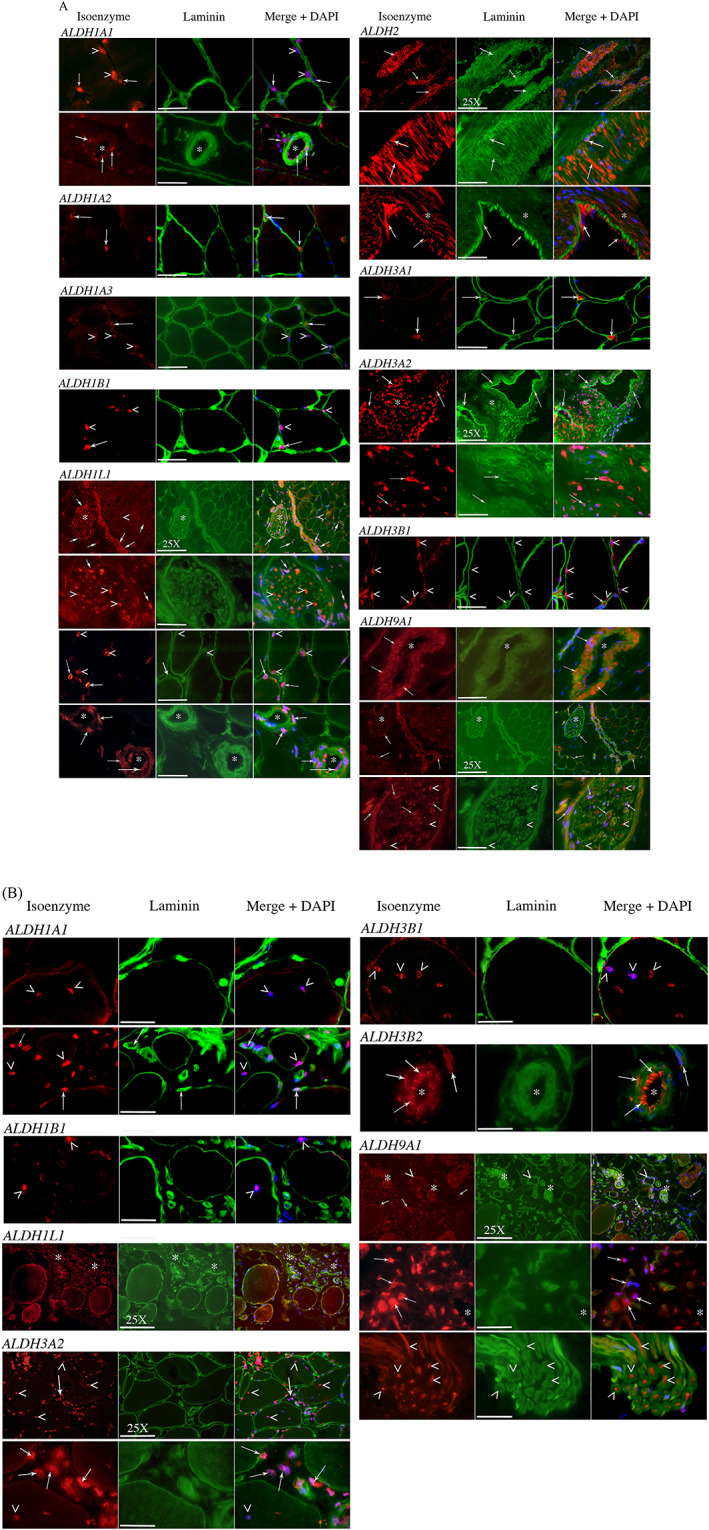
(A and B) Immunohistological localization of isoenzyme‐expressing cells *in situ* on cryostat sections. Human skeletal muscle sections were fixed with acetone and labelled for expression of several isoenzymes (red) and laminin delineating the basal lamina of muscle fibres (green). Nuclei were stained in blue with DAPI. Arrowheads point nuclear localization of isoenzymes (intranuclear, perinuclear, or both). Arrows point whole cells regardless of their localization (endothelial, interstitial, neural, myogenic, etc.). Asterisks underline global areas of interest (concentration of cells). The most representative isoenzymes are presented from healthy donors (8A), or from DMD donors (8B) when qualitative differences were perceived between the two groups. Isoenzymes were associated with myogenic‐like cells (ALDH1A1, 1B1, 1L1, and 3B1), to endothelial or angiogenic structures (ALDH1A1, 1L1, 2, 3A2, 3B2, and 9A1), to interstitial cells (ALDH1A1, 1A2, 1A3, 1L1, 3A1, 3A2, and 9A1), to neural structures (ALDH1L1 and 9A1), or to connective tissue (ALDH1L1, 3A2, and 9A1). Centronucleation is frequently observed in DMD biopsies (B) (ALDH1A1, 1B1, and 3B1). Original magnification is ×100, and it is ×25 when indicated on the scale bar. Scale bars are 40 μm (×100) and 140 μm (×25).

We identified different cell populations harbouring ALDH isoenzymes by immunohistology using snap‐frozen muscle biopsies of healthy and DMD patients (*n* = 3). Laminin staining was used to delineate the basal lamina of muscle fibres, and cells were positioned relatively to muscle fibres and basement membranes or microvasculature (*Figure* 8A and 8B).

In tissues from healthy patients (*Figure* 8A), some isoenzymes presented an intranuclear staining that covered some parts or the complete surface of the nucleus (ALDH1A1, 1A3, 1B1, 1L1, 3A2, 6A1, 8A1, and 9A1), while other isoenzymes presented mainly a perinuclear staining (ALDH1L1, 2, and 3B1). Some isoenzymes presented a cytoplasmic localization (ALDH1A2, 3A1, 3B2, and 5A1) or a nuclear and cytoplasmic localization (ALDH1L1, 2, 3A2, 8A1, and 9A1). Several nuclei were located inside the muscle fibres, in peripheral or (rarely) central position (ALDH1A1, 1A3, 1B1, 1L1, 3B1, and 8A1). Some isoenzymes labelled cells observed at positions reminiscent of that of satellite cells, that is, beneath the basement membrane and causing an excrescence to the fibre: ALDH1A1, 1A3, 1B1, 1L1, and 3B1. In the absence of the simultaneous staining of extracellular basement membrane and intracellular sarcolemma, it is not yet possible to conclude definitely regarding the status of these cells.

Some isoenzymes were expressed by cells located in the endomysial tissue: ALDH1A1, 1A2, 1B1, 1L1, 3A1, 3A2, and 3B1. Some isoenzymes were typically associated with endothelial or vascular structures (arterioles and venules): ALDH1A1, 1A2, 1A3, 1L1, 2, 3A1, 3A2, 3B2, 6A1, 8A1, and 9A1. Some tissues are especially characterized by the expression of a few isoenzymes: vessel‐associated smooth muscle cells express ALDH2, 3A2, 8A1, and 9A1; and nerve bundles strongly express ALDH1L1 and 9A1. These isoenzymes may represent new or supplementary markers of these cell types (*Table* 3). Immunostainings with antibodies directed against ALDH4A1, 5A1, 6A1, 7A1, and 18A1 were not definitely successful despite attempts using different protocols.

**Table 3:** Location of labelled structures

Location	Intranuclear		Perinuclear		Cytoplasmic		Intra‐fibre		Satellite‐like		Endomysial		Endothelial		Smooth muscle		Neural		Differences In DMD biopsies
	Control	DMD	Control	DMD	Control	DMD	Control	DMD	Control	DMD	Control	DMD	Control	DMD	Control	DMD	Control	DMD	
ALDH1A1	X some^a^	X some	X some	X some			X	X	X	X	X	X	X	X					↘^b^ Satellite‐like, rarer than in healthy patients
ALDH1A2					X						X		X						↘ Cells, rarely observed
ALDH1A3	X (some)						X		X				X rare^c^						↘ Cells, rarely observed, almost absent
ALDH1B1	X	X					X	X	X		X	X	X	X					↘ Satellite‐like and endothelial cells
ALDH1L1	X	X	X	X	X		X	X	X		X	X	X	X			X axons^d^		↘ Satellite‐like, rarer than in healthy patients
ALDH2			X	X	X most^e^	X						X	X	X	X	X			Not different
ALDH3A1					X						X		X						↘ Cells not observed, non‐specific interstitial staining
ALDH3A2	X	X			X rare	X					X	X	X	X	X	X			↗↗Much more frequent, fibrotic‐like cells
ALDH3B1			X	X			X	X	X		X	X							↘ Satellite‐like, rarer than in healthy patients
ALDH3B2					X	X							X	X					Not different
ALDH5A1					X	X	X												↘ Cells, not observed
ALDH8A1	X				X		X						X						Not observed
ALDH9A1	X	X			X	X					X	X	X	X	X		X axons	X	↘ Rare association to muscle fibres

Isoenzymes ALDH‐4A1, 6A1, 7A1, and 18A1 were absent or not detected above background.

a some: Some but not all elements in the category are stained.

b ↘: Indicates a decrease in number (qualitative assessment).

c rare: Small number of elements in the category are stained.

d axons: Within this neural tissue, axons are stained.

e most: Most elements in the category are stained.

Striking differences were noted upon examination of tissues from DMD patients (Figure 8B and *Table* 3). As expected, the DMD biopsies presented hallmarks of this myopathy, such as hypertrophic and swollen fibres, centronucleation, and the presence of abundant connective tissue. The isoenzymes presenting a nuclear localization underlined the central position of these nuclei in DMD context. Frequently, we observed an apparent decrease in the number of cells expressing isoenzymes, especially when these cells were juxtaposed to muscle fibres (ALDH1A1, 1A2, 1B1, 1L1, and 3B1) (*Table* 3). The most spectacular is the huge increase in cells expressing ALDH3A2, 1L1, and 9A1 and located in the endomysial position. These isoenzymes may, therefore, become appealing biomarkers for these cell populations that are overrepresented in the connective tissue of DMD patients.

#### 3.2.4 Expression of isoenzymes at messenger RNA level

**Figure 9:**
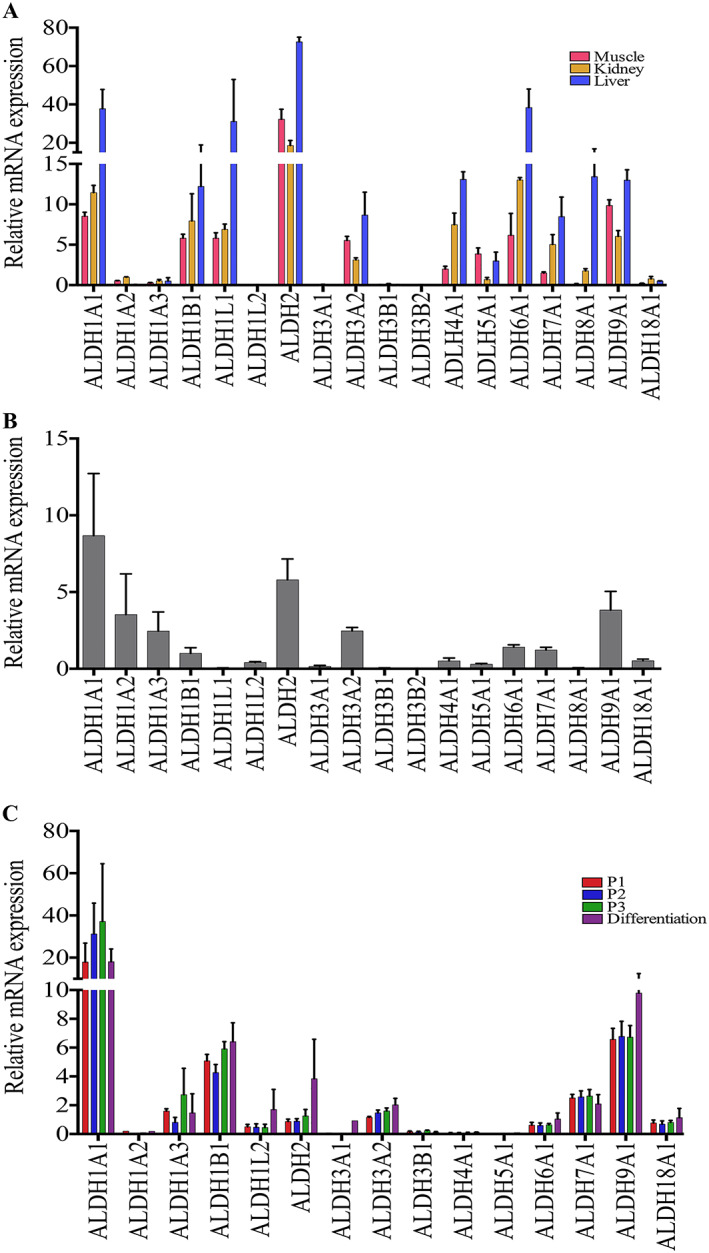
Expression of isoenzyme mRNAs in skeletal muscle tissue and cells. (A) The expression of isoenzymes was assessed and compared in human muscle (red, *n* = 2), liver (blue, *n* = 2), and kidney (yellow, *n* = 2) biopsies by Q‐PCR, underlining the presence of ALDH1A1, 1B1, 1L1, 2, 3A2, 5A1, 6A1, and 9A1. (B) The expression was analysed in the total population of freshly dissociated cells from human biopsies highlighting the presence of isoenzymes involved in the metabolism of retinoic acid (ALDH1A1, 1A2, 1A3, and 1B1) and of ALDH2, 3A2, 6A1, 7A1, and 9A1. (C) The expression was analysed and compared in primary cell cultures in proliferation and in differentiation (*n* = 4), underlining the presence of ALDH1A1, 1A3, 1B1, 2, 3A2, 7A1, and 9A1. No significant differences were observed between the proliferation and differentiation stages. Data are presented as mean and SD.

The study of isoenzyme expression at the protein level was supplemented by the quantifications of mRNAs by Q‐PCR. These were extracted from crude healthy human muscle, kidney, and liver tissues; from dissociated muscle cells; and from proliferating and differentiating cells in culture (*Figure* 9).

##### 3.2.4.1 Expression of isoenzymes *ex vivo*

Human muscle (*n* = 4), liver, and kidney (*n* = 2) were analysed concomitantly for the expression of the genes coding for 18 isoenzymes (*Figure* 9A). As expected, the liver contained the highest levels of ALDH isoenzymes, followed by the kidney. Almost all isoenzymes were also expressed in the skeletal muscle tissue, at various levels, but the profile was clearly distinct from that of liver and kidney and may serve to characterize the skeletal muscle tissue. ALDH2 is the most expressed, followed by 1A1, 1B1, 1L1, 3A2, 5A1, 6A1, and 9A1. Some isoenzymes were expressed at very low levels (1A2, 1A3, 1L2, 3A1, 3B1, 3B2, 8A1, and 18A1). The expression of ALDH16A1, which has no catalytic activity,^60^ was extremely low in all human tissues and did not change with conditions (not shown).

##### 3.2.4.2 Expression of isoenzymes by dissociated muscle cells

Just after dissociation, muscle cells (*n* = 4) mainly expressed ALDH1A1, 2, and then 9A1, 1A2, 1A3, 3A2, 6A1, 7A1, 1B1, and 4A1 (*Figure* 9B). This profile was slightly different from that observed in crude muscle, and it should be noted that most isoenzymes involved in retinoic acid metabolism were among the most expressed (1A1, 1A2, 1A3, and 1B1). Some structures and cells did not resist to enzymatic and mechanical dissociation, their isoenzyme contents have been eliminated, and the panel focused on markers of myogenic and interstitial cells.

##### 3.2.4.3 Expression of isoenzymes in cell cultures

Most isoenzymes were detected at various levels (*Figure* 9C) in these specific culture conditions prone to myogenic expansion and differentiation. ALDH1A1, 1A3, 1B1, 2, 3A2, 7A1, and 9A1 were the most expressed. ALDH2, 1A2, 1A3, 3A2, and 6A1 decreased; and ALDH1B1 and ALDH7A1 increased relatively. Some profiles evolved between proliferation and differentiation, but this did not reach statistical significance, except for ALDH9A1. ALDH1A1 presented a trend towards decrease upon differentiation, while ALDH2 presented a slight trend towards increase. ALDH3B2 and 8A1 could not be detected, but they were already weakly expressed in the other preparations. ALDH16A1 was very weakly expressed (not shown).

## 4 Discussion

This study reports the presence of ALDH‐expressing cells in healthy and dystrophic skeletal muscles as evaluated on the side of ALDEF, a convenient marker for functional exploitation of myogenic progenitors, and on the side of isoenzymes expression in view of delineating fundamental aspects of muscle homeostasis. Both sides documented the important modifications of populations in dystrophic muscles.

### 4.1 ALDEF^+^ populations remain stable with time

In healthy donors, we first observed a slight trend towards decrease with aging, which was not statistically significant. Some variations were noted between patients within the same group of age, but the clinical and physical conditions were unknown at time of hip surgery. While circulating progenitors are generally decreased with aging or in pathological conditions,^55^ our situation resembles the absence of decrease observed by Povsic *et al*.^61^ in populations of medullary cells expressing ALDH. The diversity of ALDH isoenzymes allows detoxification of several oxidative remnants, aldehydes and protein‐aldehyde adducts that progressively accumulate during aging, which all together would be especially responsible for sarcopenia,^9^, ^10^ as suggested in a variety of other models and tissues.^7^, ^24^, ^27^, ^28^, ^40^, ^62^ The diversity of isoenzymes allows metabolizing several substrates, and several isoenzymes may be expressed by a single cell population, as observed in our flow cytometry, cytofluorescence, and gene expression assays. Moreover, ALDH exerts indirect protective effects through the expression of detoxifying cascades such as glutathione peroxidase and superoxide dismutase.^8^, ^63^ Retinoic acid is diffusible and frequently acts in a paracrine fashion^64^; thus, it may diffuse from a small subset of ALDH^+^ cells and extend a protective area around small territories or niches, at least in healthy conditions. The presence of several ALDH isoenzymes with diverse detoxifying capacities in the muscle tissue suggests their protective role and may explain the persistence of ALDH^+^ cells with aging.

The proportions of ALDEF^+^ cell populations were compared between muscle groups in healthy NHP. Despite inter‐individual variabilities, we did not observe significant differences in proportions between muscle groups involved in different physiological muscular functions (positioning, breathing, jawing, and swallowing) or of different embryological origins (trunk, head, and neck). This study in NHP confirmed a trend observed previously that the lowest proportion of ALDEF^+^/CD34^−^ population relates to the paravertebral muscles.^48^ Xu *et al*.^65^ demonstrated the considerable homogeneity in satellite cell contents between muscle groups but underlined some relative differences in richness according to precise physiological requirements.

### 4.2 ALDH^+^ cell populations are imbalanced in dystrophic tissues

The situation in healthy donors contrasts strikingly with the strong increase in the proportion of ALDEF^+^ cells observed in DMD patients and GRMD dogs and suggests an important contribution to the remodelling undergoing in muscular dystrophy. ALDEF^+^/CD34^+^ cells are significantly increased in DMD patients, but we observed previously that this population was not able to regenerate the skeletal muscles *in vivo* and was associated with a mesenchymal‐like behaviour^48^ prone to adipo‐fibrotic replacement during regeneration. Indeed, a population of CD34^+^ cells is involved in adipogenesis in human skeletal muscle.^49^, ^50^

Strong links have been proposed between ALDH expression, retinoids, ALDH‐positive cells, metabolic pathways (TGFβ, NFΚb, BMP, EGF, etc.), tissue environment, proliferation, and fibrosis, although in some cases these links may follow the opposite direction.

First, in models of scarring diseases, the expression of ALDHs and the paracrine synthesis of retinoic acid dramatically trigger or exacerbate tissue fibrosis,^66^, ^67^ suggesting that the increased number of ALDH^+^ cells able to produce retinoic acid may be detrimental to appropriate regeneration or may promote fibrous scarring. Second, in some pathological tissues (e.g. skin, kidney, eyes, and liver), enhanced ALDH activity positively correlates with elevated TGFβ signalling pathway, triggering the activation and survival of fibroblasts, which overproduce ECM proteins and ultimately promote a persistent fibrotic and a pro‐inflammatory environment.^66^, ^67^, ^68^, ^69^, ^70^ In the context of DMD, TGFβ, Smad, and NFΚb are strongly involved in onset and progression of muscle fibrosis.^71^, ^72^ Also, TGFβ activates the NFΚb pathway in murine dystrophic fibroblastic cells, thus increasing their survival and proliferation.^70^ As NFΚb supports the survival and activation of macrophages, in muscle, it may be involved in the crucial phase of degeneration–regeneration that may shift from beneficial in a healthy environment to deleterious when the tissue undergoes inflammation and fibro‐adipocytic infiltration. Third, the direct effect of TGFβ on ALDH expression varies according to models. While in pancreatic cancer cells the increase in TGFβ increases Smad4 but decreases the expression of ALDH1A1,^73^ the expression of TGFβ stimulates the expression of ALDH and the production of ALDH‐positive cancer stem cells.^74^, ^75^ Taken together, these reports suggest a link between the TGFβ signalling pathway and the expression of ALDHs, leading to proliferation of fibro‐adipogenic progenitors and fibrosis, at least in the skeletal muscle.

The difference between control and dystrophic muscles was confirmed upon histological studies. We observed abundant amounts of interstitial cells expressing mainly ALDH3A2, 1L1, and 9A1 isoenzymes in DMD muscles, which could become markers of fibrogenic or fibro‐adipogenic cells especially in dystrophic conditions. It is yet unknown whether the population imbalances participate actively in the extension of the disease or reflect the steps of muscle remodelling. In DMD, a detrimental loop including ALDHs and TGFβ may be responsible for onset and then expansion of muscle fibrosis in patients.

Our results obtained in DMD patients and GRMD dogs may represent two phases of the disease. On the one hand, the biopsies from DMD patients were obtained at the time of orthosis placement, that is, when muscle regeneration has been exhausted by the cycles of the disease and a fatty fibrotic tissue is replacing the healthy one. Indeed, the non‐myogenic ALDH^+^/CD34^+^ population was the most increased. On the other hand, the biopsies from GRMD dogs were obtained from young animals, at early stages of the disease, that is, when muscle is still vigorously regenerating and the myogenic ALDH^+^/CD34^−^ population was the most increased.

It should be noted that the population of ALDEF^+^/CD34^−^ cells, which has been previously associated with myogenicity, is not drastically depleted in human patients or in GRMD dogs. This observation may be important in view of the future use of this population in therapeutic perspective; however, the actual differentiation capacities of ALDEF^+^/CD34^−^ cells extracted from healthy and dystrophic patients remain to be compared. The final role of ALDH^+^ cells and especially of the non‐myogenic ALDH^+^/CD34^+^ population in the onset and maintenance of fibrosis in DMD muscles is not elucidated. Nevertheless, the identification of pathways involved may open the avenue to therapeutic tools, such as inhibitors of NFΚb or of TGFβ pathways, or antagonists of retinoids.^72^

### 4.3 Sub‐populations are defined among ALDEF‐metabolizing cells

ALDEF metabolism alone is useful to isolate muscle cells but not sufficient as a marker of myogenicity. Some initial subsets of ALDEF^+^, such as ALDEF^+^/CD34^+^ cells, do not express myogenic capacities.^48^ Moreover, when reporting that ALDEF^+^/CD34^−^ cells are myogenic, this selects a population on a negative criterion, the absence of a marker (CD34^−^), which is less reliable and comfortable than selection on the basis of pure positive selection. These considerations mandated the search for associated markers directed against extracellular antigens. Such methodology has been developed by others to select specific myogenic cell types,^76^ but it was not based on ALDEF metabolism.

In this study, cells were dissociated using only collagenase; that is, trypsin was avoided to spare extracellular domains of some antigens. Several markers were associated with dissociated muscle cells, some of which are being linked to ALDEF^+^/34^+^ or ALDEF^+^/34^−^ populations. The markers mainly associated with ALDEF^+^/CD34^+^ were CD10, CD49e, and CD140b. The markers mainly are associated with ALDEF^+^/34^−^ were CD9, CD31, CD36, CD49a, CD49c, CD49f, CD71, CD106, CD140a, CD146, and CD184. Of these markers, CD9, CD49c, CD49f, CD106, CD146, and CD184 have been previously associated with cells presenting myogenic capacities, whether myogenic or perivascular.^49^, ^77^, ^78^, ^79^, ^80^, ^81^, ^82^, ^83^, ^84^ The association between CD56, which is a marker of human satellite cells and myoblasts, and ALDEF, was variable at the time of dissociation. The CD56 expression became constant upon culture in myogenic condition.

When we compared the myogenic capacities in cultures of populations selected on CD56, CD9, CD49e, or CD10 markers in association with ALDEF, these populations could be ranked, in decreasing order of myogenicity, as CD56^+^ > CD9^+^ > CD49e^+^ > CD10^+^. Therefore, double‐positive selections allow preparing cell populations, in a way that may be further amenable to preclinical and clinical applications, using FACS or magnetic bead selection. In myogenic cultures, most cells exhibited both high ALDEF metabolism and expression of CD56, thus confirming the role of ALDH isoenzymes in myogenesis. This was confirmed later on by the expression of several ALDH genes in culture by Q‐PCR.

While it was first designed to allow identification and quantification of ALDH1A1^+^ cells,^3^ ALDEF can actually be metabolized by several isoenzymes depending upon the tissue and cell population under investigation. For example, the metabolism by ALDH2 and ALDH1A2 has been observed in leukaemia and lung cancer cells overexpressing these enzymes.^14^ The presence of ALDH1A1, 1A3, 2, 4A1, 5A1, 6A1, 7A1, and 18A1 has been observed in ALDEF‐positive sorted cells.^13^ Of note, the DEAB used to block ALDEF metabolism and provide a negative control does not present the same selectivity towards all isoenzymes,^85^ and the isoenzymes do not present the same enzymatic constants. We frequently observed differences between the percentages of cells expressing a given isoenzyme, and the total percentage of cells metabolizing ALDEF, and this was especially striking regarding ALDH2 and ALDH3A1. On the one hand, this may indicate that in muscle cells, some isoenzymes are present but do not participate in the metabolism of ALDEF in the condition of the assay. On the other hand, the final percentage of ALDEF staining may not reflect post‐translational modifications of some isoenzymes^59^ or competition between isoenzymes with differing enzymatic constants for the substrate, or the different levels of blockade by DEAB. There is no doubt that the use of ALDEF reagent allows selecting populations of myogenic cells; however, the exact nature of isoenzymes able to metabolize ALDEF is not clearly known. Therefore, we attempted to complete the landscape of muscle isoenzymes in the skeletal muscles using complementary methodologies.

### 4.4 Several isoenzymes are observed in muscle tissue and muscle cells by histology

Several isoenzymes were identified in different cell populations and structures using histology. Some populations were located close to muscle fibres in position resembling that of myogenic satellite cells (ALDH1A1, 1B1, and 1L1). Some populations were located in positions reminiscent of endothelial capillaries (ALDH2, 3B2, and 9A1), or associated with small venules or arterioles (ALDH1A1, 2, 3B2, and 8A1). Some populations were located in the endomysium (ALDH1A1, 2, 3B1, and 7A1), and we already noted that some of them were exacerbated and may be involved in the formation of connective tissue in dystrophic muscles (ALDH1L1, 3A2, and 9A1). Finally, some isoenzymes were expressed by neural structures (ALDH1L1 and 9A1). Some isoenzymes therefore show multiple localizations and should be linked to different cell fates or functions: ALDH1A1 (myogenesis, angiogenesis), ALDH1L1 (myogenesis, angiogenesis, and neurogenesis), and ALDH9A1 (angiogenesis, neurogenesis, and formation of connective tissue).

Frequently, isoenzymes labelled nuclei, preferentially inside muscle fibres (ALDH3A1 and 3B1) and also outside the fibres (9A1) or both (3A2). A nuclear localization has been previously observed with ALDH3A1 and 7A1 isoenzymes, where it would directly participate in cell cycle regulation, resistance to DNA damage, and facilitation of repair and reduction of apoptosis.^2^ Nuclear localizations therefore suggest unique roles in muscle cell physiology played by some ALDH isoenzymes.

In muscle cell cultures, several individual isoenzymes are expressed, as assessed by cytofluorescence, suggesting roles in proliferation, regeneration, and differentiation. A similar trend has been observed in several tissues by others (i.e. pancreatic cells, cardiac cells, and haematopoietic cells).^32^, ^43^, ^46^ In conditions favouring myogenicity, ALDH1A1, 1A3, 1B1, 3A2, 7A1, and 9A1 are the most prominent during proliferation and may constitute a signature of myogenesis.

### 4.5 Isoenzyme genes are differentially expressed in distinct muscle contexts

The skeletal muscle has been rarely investigated for expression of ALDH genes. Recently, Terry *et al*.^86^ performed an extensive RNASeq analysis of muscle types in rodents. They reported the expression of all ALDH isoenzymes, at various levels depending on the isoenzyme. ALDH1A1 was the most expressed, followed by ALDH2 and 4A1. Interestingly, for a given isoenzyme, some variations were noted in the level of expression between anatomical and functional muscle groups. To obtain complementary follow‐up, we achieved gene expression studies in three different situations: in crude skeletal muscle tissue, in muscle cells obtained after tissue dissociation and elimination of fibres, and in primary cultures. Some discrepancies were noted between the results of protein detection (cytofluorimetry, cytofluorescence, and immunohistology) and mRNA expression (Q‐PCR) that may be due to the nature and sensitivities of the reagents and methodologies. For example, ALDH1B1, 2, 4A1, 5A1, 6A1, and 18A1 and to some extent 7A1 are mainly located in the mitochondrial compartment, not in cytosol,^17^ and may be more difficult to identify in histology. It was also reported recently that the process of tissue dissociation itself activates some panels of genes within skeletal muscle cells, which may also create discrepancies between results obtained using different methodologies and cell status.^87^ Nevertheless, overlapping of the results was most frequently encountered, at least on the qualitative point of view.

In crude human skeletal muscle tissues, isoenzymes ALDH1A1, 1A2, 1A3, 1B1, 1L1, 2, 3A1, 3A2, 7A1, and 9A1 were identified by both histological and Q‐PCR methodologies, although at lower levels by histology regarding ALDH7A1. Our data confirm some initial northern blot expression studies that reported the presence in crude skeletal muscle tissue of ALDH1A1,^88^ 1L1,^89^ 2,^88^ 3A2,^90^ 4A1,^91^ 5A1,^92^ 9A1,^93^ and 18A1.^94^ At variance, we also observed the expression of ALDH1A3, 1B1, and 6A1, likely because of methodological refinements since the early description or owing to the difference in tissue source.

In dissociated muscle cells, ALDH1A1, 1A2, 1A3, 2, 3A2, 3B2, 4A1, 7A1, and 9A1 were identified by both cytofluorimetry and Q‐PCR methodologies. ALDH1A2 and 1A3 were increased in dissociated cells compared with crude skeletal muscle, suggesting that these isoenzymes are more abundant in mononucleated cells than in muscle fibres, which are destroyed by the dissociation. Conversely, ALDH1L1, 2, 3A1, and 3B1 are decreased suggesting that these isoenzymes are involved in larger muscle structures; indeed, ALDH1L1 and 2 are observed within vessels, capillaries, and neural superstructures at the histological level, which may not be spared by the dissociation process.

Primary cultures were highly enriched in myogenic progenitors expressing CD56 and contain ALDH1A1, 1A3, 1B1, 2, 3A2, 7A1, and 9A1 as identified by cytofluorescence and Q‐PCR. These results suggest that several isoenzymes are involved in myogenesis and/or cell survival *in vitro*. Other cell types have been reported to expand while keeping expression of ALDEF and a progenitor status.^32^, ^46^, ^95^, ^96^ ALDH1A1 was expressed in our primary muscle cultures as previously described, but we also noticed the strong increase in ALDH1B1 and the moderate increases in ALDH7A1 and 9A1 in culture when compared with isolated mononucleated cells, suggesting that these isoenzymes may be positively involved in myogenesis *in vitro*. Meanwhile, ALDH2 was decreased and ALDH1A2 was almost abolished in culture; they may not be specifically involved in myogenesis but rather in homeostasis of other cell types.

Little is known regarding the sequential involvement of isoenzymes during myogenesis. Retinoic acid is involved,^19^, ^20^, ^21^, ^23^, ^97^, ^98^, ^99^ and several isoenzymes participating in its production and regulation are expressed in muscle as presented here (ALDH1A1, 1A3, 1B1, and 8A1) and may have additive or compensating effects as illustrated in other systems using knock‐out animal models.^18^, ^100^, ^101^

Specific roles of some isoenzymes have been pointed in dedicated tissues, but the skeletal muscle has been rarely investigated if any (except for northern blot analysis several years ago). ALDH1A1 is involved in several embryological pathways such as formation of neural, haematopoietic, or cardiac tissues and myogenesis.^25^, ^26^, ^27^, ^28^, ^29^ ALDH1A3 is involved in cardiopulmonary system formation during embryogenesis and in the formation and maintenance of ocular systems. ALDH1B1 is involved in the formation, maintenance, and expansion of pancreatic progenitors^32^ and subsets of pancreatic cells.^95^ Whether a parallel can be drawn between its unique role in pancreas development and potential roles in the skeletal muscle suggested by our studies is unknown yet. ALDH2 is involved in vascular adaptation, reactivity, and protection of large vessels against ischaemia.^38^ In our study, cells expressing ALDH2 are mainly located inside vessel walls, or in positions reminiscent of that of endothelial cells. The expression of ALDH2 by endothelial cells would explain the high proportion of ALDH2^+^ cells extracted from muscle biopsies and the relative decrease in their proportion in culture under myogenic conditions. ALDH7A1 is involved in the formation of zebrafish eyes and fins^39^ through the proliferation of progenitors, together with ALDH2 and 1A2.^102^ All these isoenzymes are expressed in various types of cancers through the participation in survival, maintenance, and proliferation of cancer stem cells.

### 4.6 Attempt for a synthetic overview

Gathering the information brought by our tools, we may propose some preferential roles for the different ALDH isoenzymes. We essentially considered their histological localization, the variations in mRNA expression (from crude muscle, dissociated mononucleated cells, and cells grown in a myogenic specific medium), and their expression *in vitro* upon culture in a myogenic medium.

ALDH1A1, 1A3, 1B1, 1L1, and 9A1 would be involved in general homeostasis combining myogenesis and angiogenesis. Of these, ALDH1L1 and 9A1 would be also involved in neural support in the tissue. ALDH1A2, 2, 3A1, 3B2, and 8A1 would be preferentially involved in angiogenesis or endothelial homeostasis. ALDH3A2 would be involved in stromal support. ALDH3B1 and 7A1 would be preferentially involved in myogenic homeostasis. The involvement of ALDH4A1, 5A1, 6A1, 16A1, and 18A1 could not be established, either because of a complete lack of function in muscle or because of inadequacy of tools. ALDH3A2 and ALDH9A1 would be especially involved in the physiopathological process of DMD, because of their increased presence in the fibrotic tissue.

Taken together, our results suggest that, supplementary to ALDH1A1 that was previously described, several isoenzymes present in both myogenic and non‐myogenic cells take part in skeletal muscle homeostasis *in vivo* and *in vitro* and would help in defining an identity card of this tissue. Minimally, ALDH1A2, 1A3, 1B1, 1L1, 2, 3A2, 3B1, 7A1, 8A1, and 9A1 deserve future individual investigations, given their high expression level, their association to muscle structures and cells, and their biochemical detoxifying functions.

### 4.7 Limitations of the study and perspectives

This study was hampered by some technical or methodological limitations, represented by the variability of the muscle samples extracted from human patients with intrinsically variable status. Limitations were also met using some antibodies showing variable reactivities between methodologies (flow cytometry, immunohistology, and cytofluorescence). The present study, however, is the first to assess the presence of the ALDH isoenzymes family in the skeletal muscle *in situ* and *in vitro*. Hence, through their presence within several populations in the tissue, their expression in native condition and in culture, their distribution and their persistence with aging, and their imbalance in dystrophic conditions, ALDH isoenzymes are proposed to play roles in skeletal muscle homeostasis. These isoenzymes may therefore constitute new therapeutic targets for pharmacological, molecular, or genetic modulations. Some of them may also define a panel of new specific markers of muscle cell populations. Furthermore, ALDEF^+^ cell populations, in association with dedicated markers, may constitute new promising candidates for regenerative therapy approaches.

## 5 Acknowledgements

This work was supported by the DIM‐STEMPOLE from Région Ile‐de‐France (J.E.), the Association Française contre les Myopathies (AFM; C.C., S.R., and J.L.), and by grants from the Duchenne Parent Project from the Netherlands (DPP), from the Ligue contre la Cardiomyopathie, and from the Agence de Biomédecine. We thank M. Stéphane Vasseur and Ms. Maud Chapart, and MYOBANK‐AFM (the AFM tissue bank for research, BB‐0033‐00012, Paris, France), for their invaluable help and expertise in providing human muscle biopsies. We thank Dr. Veronique Sazdovitch for the providing the human kidney and liver tissues (Departement de Neuropathologie R. Escourolle, Paris, France). We thank Dr. Chantal François and Mrs. Elodie Laffrat (Institut du Cerveau et de la Moelle, Paris, France), Dr. Marjorie Lagrevol and Dr. Kevin Thibault‐Duprey (SANOFI, Alfortville, France), Dr. Claire‐Maëlle Fovet (MIRCen‐CEA, Fontenay‐aux‐Roses, France), and Dr. P. Pradeau (IMASSA, France) for the kind gift of biopsies from euthanized macaques. We thank Dr. Inès Barthélémy (École Nationale Vétérinaire d'Alfort, ENVA) for the kind and invaluable gift of biopsies from healthy and GRMD dogs. We thank Mrs. Catherine Blanc and Mrs. Brigitte Hoareau‐Coudert (Flow Cytometry Core CyPS, Pierre & Marie Curie University, Pitié‐Salpêtrière Hospital, Paris) for invaluable help in cell sorting, and Dr. Valérie Vanneaux (Cell Therapy Laboratory, Saint Louis Hospital, Paris) for her FACS expertise. We thank Drs. Valérie Allamand, Sonia Berrih‐Aknin, Raphaëlle Grifone, Martine Oloko, and Frédérique Truffault for their expert skills, advice, and discussions.

The authors certify that they comply with the ethical guidelines for authorship and publishing of the *Journal of Cachexia, Sarcopenia and Muscle*. 103

## 6 Conflict of interest

The authors declare no competing interests.
